# A multi-centre, cross-sectional study on coronavirus disease 2019 in Bangladesh: clinical epidemiology and short-term outcomes in recovered individuals

**DOI:** 10.1016/j.nmni.2021.100838

**Published:** 2021-01-08

**Authors:** A. Mannan, H.M.H. Mehedi, N.U.H.A. Chy, Md. O. Qayum, F. Akter, M.A. Rob, P. Biswas, S. Hossain, M. Ibn Ayub

**Affiliations:** 1) Department of Genetic Engineering & Biotechnology, Faculty of Biological Sciences, University of Chittagong, Chattogram, Bangladesh; 2) Department of Medicine, 250 bedded General Hospital, Chattogram, Bangladesh; 3) Health Economics Research Group, Department of Economics, University of Chittagong, Chattogram, Bangladesh; 4) Curator, Institute of Epidemiology, Disease Control & Research (IEDCR), Dhaka; 5) Department of Endocrinology, Chittagong Medical College, Chattogram, Bangladesh; 6) Department of Pathology, Mymensingh Medical College, Mymensingh, Bangladesh; 7) Corona Unit, Dhaka Mohanagar General Hospital, Dhaka, Bangladesh; 8) Department of Genetic Engineering & Biotechnology, University of Dhaka, Ramna, Dhaka, Bangladesh

**Keywords:** Asymptomatic, Bangladesh, comorbidities, coronavirus disease 2019, epidemiology, post-coronavirus disease 2019 complications

## Abstract

Coronavirus disease 2019 (COVID-19) rapidly became a global pandemic. This study aimed to investigate severe acute respiratory syndrome coronavirus 2 (SARS-CoV-2) -associated epidemiology and clinical outcomes in Bangladesh in order to understand the future course of the COVID-19 pandemic and develop approaches to prevention. A cross-sectional study based on retrospective interviews was conducted on 1021 individuals with RT-PCR-confirmed COVID-19 admitted in six different hospitals in Bangladesh and who recovered 4 weeks before the interview date. Of the 1021 patients, 111 (10.9%) were asymptomatic and the other 910 (89.1%) were symptomatic. Higher prevalence of COVID-19 was found in the male population (75%), in cohorts with B-positive blood group (36.3%) and in the 31–40 years age group. Common symptoms observed in our study participants were fever (72.4%), cough (55.9%), loss of taste (40.7%) and body ache (40%); whereas among the biochemical parameters, neutrophil count (46.4%), D-dimer (46.1%) and ferritin (37.9%) levels were elevated. Among the recovered individuals, short-term outcomes including pains and aches (31.8%), weakened attention span (24.4%) and anxiety or depression (23.1%) were also significantly prevalent in the symptomatic cases with comorbidities. Our study showed that in Bangladesh, adult males aged between 31 and 40 years were more vulnerable to developing COVID-19. It also indicated a rising trend of asymptomatic cases as the pandemic progressed. As a consequence, deployment of interventions to curb further spread of community infection is necessary to avoid grave outcomes of COVID-19 in Bangladesh.

## Highlights

• 10.9% COVID-19 patients were found to be asymptomatic.
• Comorbid patients were having significantly more post-COVID-19 complications.
• The most prevalent symptoms were fever (72.4%), and cough (55.9%).
• Most common post-recovery complication was pains and aches (32%).
• Diabetes was the most common comorbidity among COVID-19 patients.

## Introduction

Coronavirus disease 2019 (COVID-19) is a contagious disease of the respiratory system [1]. In terms of genetic characteristics, its causal agent, severe acute respiratory syndrome coronavirus 2 (SARS-CoV-2), is significantly diverse from SARS-CoV and Middle East respiratory syndrome (MERS) -CoV [2,3]. The virus's rapid progression around the globe has unfolded a variety of clinical manifestations in different geographical locations. Severe prognosis of COVID-19 has been reported to be associated with comorbidities including diabetes, hypertension, cardiovascular disease, chronic obstructive pulmonary disease, malignancy, and chronic liver disease [4].

With a population of more than 161 million people, Bangladesh stands eighth among the most populated countries in the world [5]. In Bangladesh, as of 9 October 2020, infections from SARS-CoV-2 had spiked to nearly 375 870 individuals and the death count was 5477 people (https://iedcr.gov.bd/). With regard to the clinical outcomes of COVID-19 and its association with various pathophysiological factors, no comprehensive study has been conducted in Bangladesh. Some of the studies did not include a large sample size [[6], [7], [8]], so understanding the aforementioned relationship of COVID-19 symptoms with comorbidities and biochemical parameters from previous studies was not possible. In particular, the long-term complications or short-term outcomes among patients who have recovered from COVID-19 have not been studied with the necessary rigor. Investigating the epidemiological characteristics of individuals diagnosed with COVID-19 in Bangladesh will help to provide a proper insight into the clinical characterization and patterns in progression of the disease. It is vital to examine these aspects and factors related to the outcomes of COVID-19 to enact appropriate means of prevention and treatment.

The present study uses a comprehensive approach for the epidemiological characterization of individuals with COVID-19 in Bangladesh; we managed to create a large data set for our study by collecting information for a consecutive 3 months during the pandemic. We aimed to investigate the patterns and array of symptoms in individuals with confirmed COVID-19 as well as assess their relationship with the presence of comorbidities and results from biochemical assays with various other preliminary short-term clinical findings post recovery.

## Materials and methods

### Study design

A cross-sectional retrospective study was conducted among COVID-19-positive patients confirmed by RT-PCR using both nasopharyngeal and oropharyngeal swabs as clinical specimens. These COVID-19-diagnosed patients were admitted in six different hospitals in Bangladesh. Post-COVID-19 clinical characteristics was recorded upon each individual's discharge from the hospital and after confirming that these individuals were indeed COVID-19 free by conducting two consecutive RT-PCR assays 24 hours apart. Recovered patients (according to negative RT-PCR result) at least 4 weeks before the interview were considered for this study. We also categorized all positive patients into two categories (symptomatic and asymptomatic) according to the presence of any one of the established symptoms described by WHO [9] and CDC (https://www.cdc.gov/coronavirus/2019-ncov/symptoms-testing/symptoms.html). The RT-PCR test was performed using the Novel Coronavirus (2019-nCoV) Nucleic Acid Diagnostic Kit (PCR-Fluorescence Probing) by Sansure Biotech Inc. (Changsha, China) in Institute of Epidemiology Disease Control and Research (IEDCR) approved laboratories in Bangladesh.

### Study sites and sample size

The study was conducted in six hospitals with specialized units (following government instructions) for isolating and treating individuals with COVID-19 from two different divisions of Bangladesh namely Dhaka (Dhaka Mohanogor General Hospital and Narayanganj Sadar Hospital) and Chattogram (Chittagong General Hospital, Chittagong Medical College Hospital, Chattogram Field Hospital and Chandpur Sadar Hospital). The study period started on 1 April 2020 and ended on 30 June 2020.

### Data collection and case enrolment

The physicians collected data relevant to the study retrospectively by interviewing 1021 COVID-19 patients over the telephone and all data were recorded into a preset Google form approved by the institutional review board of the 250-bed Chattogram General Hospital. After diligent verification, the google forms were submitted and stored in our secured database. Medical prescriptions and hospital records were also accessed and matched with patient data obtained through the interview. Questionnaires included patient's sociodemographic information, clinical manifestations, biochemical parameters, behavioural practice, comorbidities, medications, vital signs, laboratory tests, electrocardiogram results, inpatient medications, treatments and outcomes (including length of stay, discharge, re-admission and mortality).

### Exclusion criteria

Deceased patients and those who were not interested in participating or did not give consent to data collection and usage for research purposes were not included.

### Ethical consideration

The protocol was approved by the Institutional Review Board of the 250-bed General Hospital, Chattogram, Bangladesh (IRB#00981).

### Statistical analysis

Descriptive statistical analyses were performed expressing categorical variables with numbers and proportions. They were compared using χ^2^ test and Fisher's exact test. Values of p less than or equal to 0.05 (two-sided) were considered statistically significant. Multivariate logistic regressions were performed to identify the predictors of long-term post-COVID complications. All statistical analyses were performed using Stata/MP 14.0 (StataCorp, College Station, TX, USA) and GraphPad Prism (GraphPad, San Diego, CA, USA). The study considered COVID patients showing no symptoms of infection as asymptomatic and those showing at least one of the typical symptoms as symptomatic. Similarly, patients with at least one type of comorbidity were considered comorbid and those with no comorbidity were considered non-comorbid patients.

## Results

### Demographic information and vaccination history of COVID-19 patients in the study

Of the 1021 COVID-19 inpatients studied, a total of 111 (10.9%) were found to be asymptomatic, and the number of symptomatic cases was 910 (89.1%) (Table 1).

**Table 1:** Demographics and baseline characteristics of patients diagnosed with COVID-19

	All patients (*n* = 1021)	Asymptomatic (*n* = 111)	Symptomatic (*n* = 910)	p value^a^
	*n* (%)	*n* (%)	*n* (%)	
Age (years)				
0–9	18 (1.8)	8 (7.2)	10 (1.1)	0.000
10–19	50 (4.9)	8 (7.2)	42 (4.6)	
20–29	248 (24.4)	32 (28.8)	216 (23.8)	
30–39	309 (30.4)	32 (28.8)	277 (30.5)	
40–49	171 (16.8)	11 (9.9)	160 (17.6)	
50–59	126 (12.4)	15 (13.5)	111 (12.2)	
60 and above	96 (9.4)	5 (4.5)	91 (10)	
Gender				
Male	764 (75)	72 (64.9)	692 (76.1)	0.010
Female	256 (25)	39 (35.1)	217 (23.9)	
BMI (kg/m^2^)				
<18.5	20 (2.1)	4 (4)	16 (1.9)	0.475
18.5–22.9	258 (27.4)	29 (29.3)	229 (27.2)	
23.0–26.9	434 (46.1)	43 (43.4)	391 (46.4)	
27.0 and above	230 (24.4)	23 (23.2)	207 (24.6)	
Blood group				
A+	207 (20.9)	27 (25)	180 (20.4)	0.009
A–	12 (1.2)	1 (0.9)	11 (1.2)	
AB+	92 (9.3)	4 (3.7)	88 (10)	
AB–	10 (1)	4 (3.7)	6 (0.7)	
B+	360 (36.3)	34 (31.5)	326 (36.9)	
B–	15 (1.5)	2 (1.9)	13 (1.5)	
O+	287 (29)	33 (30.6)	254 (28.7)	
O–	9 (0.9)	3 (2.8)	6 (0.7)	
Vaccinated	903 (90.4)	96 (88.9)	807 (90.6)	0.575
Having BCG mark	835 (84.7)	93 (89.4)	742 (84.1)	0.156
Comorbidities				
Diabetes	197 (19.3)	14 (12.6)	183 (20.1)	0.058
Cancer	10 (1)	2 (1.8)	8 (0.9)	0.298
Cardiovascular disease	78 (7.7)	7 (6.3)	71 (7.8)	0.573
Respiratory disease	86 (8.4)	5 (4.5)	81 (9)	0.146
Liver disease	20 (2)	0 (0)	20 (2.2)	0.154
Kidney disease	27 (2.7)	3 (2.7)	24 (2.6)	1.000
Other chronic disease	80 (7.8)	11 (10)	69 (7.6)	0.391

Abbreviations: BCG, bacillus Calmette–Guérin; BMI, body mass index; COVID-19, coronavirus disease 2019.

a Fisher's exact test for categorical variables was used in analysing p values. p < 0.05 was considered statistically significant.

**Fig. 1:**
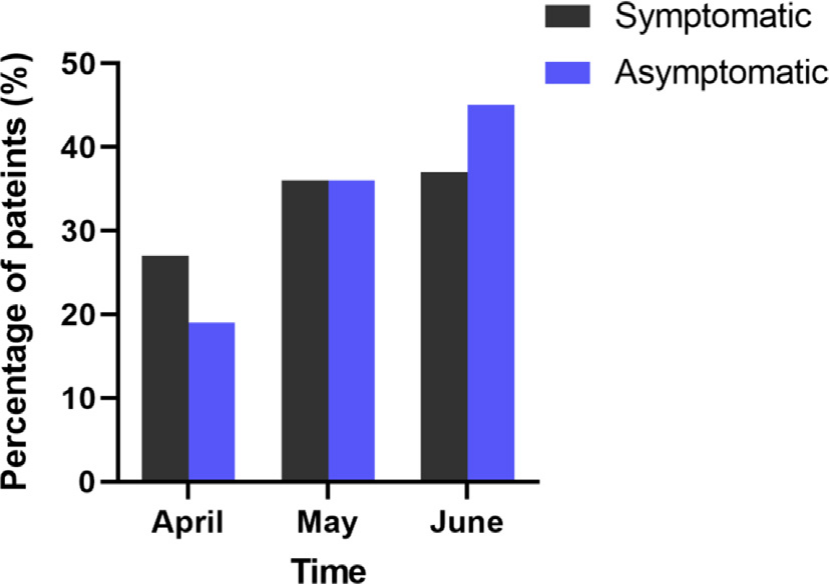
Frequency of symptomatic and asymptomatic coronavirus disease 2019 patients at various time-points.

As shown in Table 1, an individual's age was found to have an association with being asymptomatic or symptomatic (p 0.000). Higher proportions of asymptomatic cases were found in the age groups of 0–9 (7.2%), 10–19 (7.2%), and 20–29 (28.8%) years. Common to both symptomatic and asymptomatic groups, the proportion of males was greater than that of females, the values being 64.9% and 76.1%, respectively (p 0.01). Individuals in the symptomatic group showed more likelihood towards being overweight (46.4%) and obese (24.6%). A significant bivariate relationship was found (p 0.009) between blood group and a patient's being symptomatic or asymptomatic. More than 90% of all patients had received all required basic vaccines, and almost 85% had a clear bacillus Calmette–Guérin scar on their arms. Diabetes was the most prevalent comorbid condition (19.3%), followed by respiratory (8.4%) and cardiovascular (7.7%) diseases among the study participants. An over time trend in asymptomatic and symptomatic COVID-19 was also examined. Fig. 1 clearly illustrates an upward trend among asymptomatic COVID-19 cases with time, whereas the trend for symptomatic cases remained more or less similar.

### Clinical manifestations of symptomatic COVID-19 patients

Table 2 presents counts and proportions of usual signs and symptoms as observed in individuals diagnosed with COVID-19 presenting with one or more such symptoms. Of the symptomatic cases, a total of 458 (52%) patients experienced a body temperature exceeding 38.3°C, whereas for 357 (40.75%) patients body temperature ranged from 37.2°C to 38.3°C. The oxygen saturation rate went below 90% for 19.6% of the patients, and another 8% of patients experienced a low saturation level of 90% to 92.9%. The systolic and diastolic blood pressure measures were found to be greater than 159 mmHg and 99 mmHg, respectively, for 5% of the symptomatic cases. The most prevalent symptoms among the cases in order of prevalence rate were fever (81%), cough (62.7%), loss of taste (45.66%), body ache (44.77%), loss of smell (41%), breathing difficulty (29%) and sore throat (26.62%).

**Table 2:** Signs and symptoms of individuals with coronavirus disease 2019 (symptomatic)

Characteristic	*n*	%
Body temperature		
<37.2°C	61	6.96
37.2°C–38.3°C	357	40.75
>38.3°C	458	52.28
Oxygen saturation rate		
<90%	78	19.60
90%–92.9%	32	8.04
93%–95.9%	130	32.66
>96%	158	39.70
Blood pressure		
<120/<80 mmHg	85	10.30
120–140/80–89 mmHg	693	84.00
141–159/90–99 mmHg	5	0.61
>159/>99 mmHg	42	5.09
Symptoms		
Fever	738	81.19
Cough	570	62.71
Taste loss	415	45.65
Smell loss	376	41.36
Breathing problem	264	29.04
Sore throat	242	26.62
Diarrhoea	202	22.22
Running nose	190	20.90
Chest pain	178	19.58
Tiredness	127	13.97
Vomiting	116	12.76
Conjunctivitis	40	4.40

### Biochemical parameters of COVID-19 patients

**Fig. 2:**
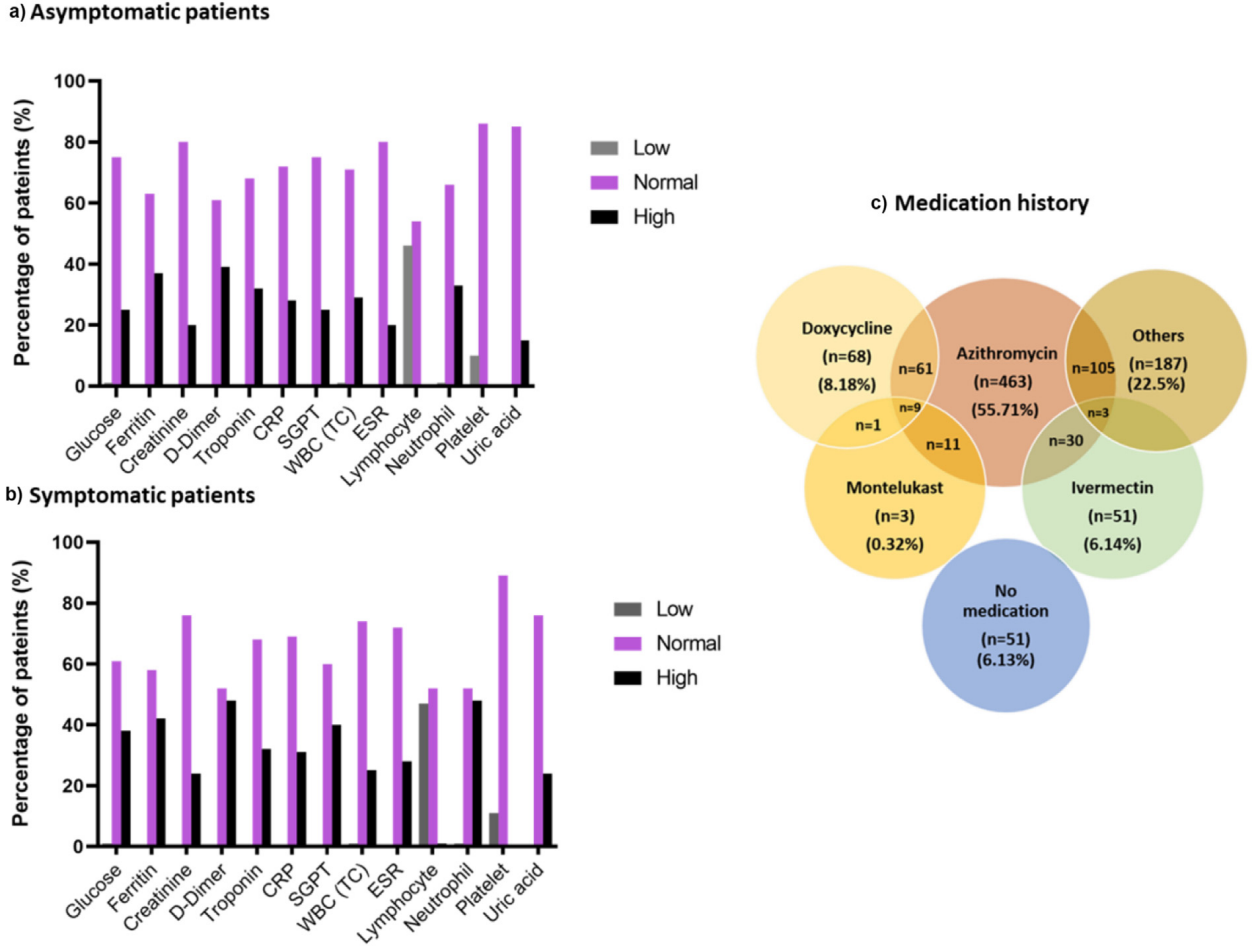
Biochemical parameters and medication history of coronavirus disease 2019 (COVID-19) patients. (a) Various biochemical measurements of asymptomatic patients during onset of COVID-19. Normal reference values are: D-dimer <0.5 μg/mL; Ferritin male 18–370 ng/mL, female 9–120 ng/mL; serum creatinine male 0.4–1.4 mg/dL, female 0.3–1.1 mg/dL; C-reactive protein (CRP) <5 mg/L; troponin <0.30 ng/mL; uric acid male 3.4–7 mg/dL, female 2.4–6 mg/dL; erythrocyte sedimentation rate (ESR) male <22 mm/h, female <29 mm/h; alanine transaminase (SGPT) male: 15–65 U/L, female 15–60 U/L; white blood cell count (WBC) 4 × 10^9^–11 × 10^9^/L; neutrophils 40%–75%; lymphocytes 20%–45%. (b) Various biochemical measurements of symptomatic patients during onset of COVID-19. (c) Medication history of the patients during the tenure of COVID-19 persistence for cases.

For a substantial number of participants in this study, biochemical assay results were found to deviate from the normal range for each of the markers measured in the analyte (Fig. 2A). Neutrophils and D-dimer were found to be high for 45.51% and 46.01%, respectively, of the patients presenting with symptoms, whereas for approximately 37%, the diagnostic tests showed a high level of blood glucose, ferritin, C-reactive protein and alanine transaminase. Among other parameters, troponin and erythrocyte sedimentation rate were high for 28.38% and 26%, respectively, of the patients, whereas creatinine, and total white blood cell count were elevated for around 25% of the symptomatic patients. A low lymphocyte count was found for 3% of the patients and laboratory test results for 7.43% of the patients reported low platelet count as well. Among the asymptomatic patients, around 40% had high levels of ferritin and D-dimer. Troponin and neutrophils were high for about 30%, glucose, alanine transaminase, C-reactive protein and total white blood cell count for 25%, Creatinine and erythrocyte sedimentation rate for 20% and uric acid for 15%. Almost 50% of the asymptomatic patients were found to have a low level of lymphocytes and about 10% had a low platelet count.

### Medication history during the tenure of COVID-19 persistence for cases

Individuals with COVID-19 during the tenure of their SARS-CoV-2-positive status took some day-to-day medications along with antibiotics (Fig. 2B). For the antibiotics, 55.71% took azithromycin alone, and became 74% in total when it was consumed in combination with other therapeutic drugs establishing a higher proportion in terms of intake compared with other frequently taken antibiotics. Doxycycline was taken alone by 8.18% of the patients, whereas for ivermectin the sole consumption and consumption in combination stood at 6.14% and 8.2%, respectively. Of the other types of medications, Montelukast was taken by 0.3%, and 22.5% of the patients took multiple other types of therapeutic drugs. Only 6.13% of the patients did not take any medications during COVID-19.

### Preliminary short-term outcomes and health issues in individuals recovered from COVID-19

The most prevalent post-recovery complications that were reported included sleep disturbance (32%), pains and aches (31.8%), weakened attention span (24.4%), anxiety and depression (23.1%), memory loss (19.5%), and complications with mobility (17.7%) (Table 3). Considering comorbidity as a determinant, χ^2^ tests for post-recovery complications returned significant deviations between individuals with comorbidities and those without for all reported complications but panic attacks. Comorbid patients were found to be more likely to experience mobility problem (26%), weakness and problems performing usual activities (14%), pains and aches (40%), anxiety and depression (28.5%), sleep disturbances (41.3%), concentration loss (28.5%), and memory loss (24.6%) than those without any comorbid conditions. There were significant differences between the two groups for all complications except panic attacks. The Supplementary material (Table S3) contains estimated results from logit models designed for post-recovery complications. As the adjusted odd ratios indicate, the respiratory disease caused each outcome of memory loss and pain and aches at 5% level and sleep disturbance at 1%. Patients receiving medication for a long time significantly increased the probability of loss of memory, loss of concentration and having sleep disturbance at 1% level and anxiety and depression at 5% level compared with those not taking medications for long periods. A comparison of the patients, based on the most prevalent blood groups and post-COVID complications is presented in the Supplementary material (Table S4).

**Table 3:** Post-coronavirus disease 2019 complications of patients by comorbidity status

	All patients (*n* = 1021)	With comorbidity (*n* = 358)	Without comorbidity (*n* = 662)	*P* value
Mobility problem	179 (17.7)	93 (26)	86 (13)	0.000
Routine-activity weakness problem	104 (10.7)	50 (14)	54 (8.2)	0.003
Pains and aches	319 (31.8)	143 (40)	176 (26.6)	0.000
Anxiety and depression	230 (23.1)	102 (28.5)	128 (19.3)	0.000
Sleep disturbances	312 (32)	148 (41.3)	164 (24.8)	0.000
Panic attack	121 (12.4)	43 (12)	78 (11.8)	0.712
Weakened attention span	243 (24.4)	102 (28.5)	141 (21.3)	0.005
Memory loss	195 (19.5)	88 (24.6)	107 (16.2)	0.001

### Contact history of the COVID-19 patients in this study

This study also assessed the contact history of the participating patients, focusing on multiple variables representing direct and indirect contacts (see Supplementary material, Table S1); 50% of the diagnosed cases came into close contact with other COVID-19-positive individuals, and 40.6% of the total participants had family members who were diagnosed with COVID-19. A total of 608 (62%) patients reported having an indirect contact, and 466 (48.5%) patients made frequent visits outside their home before being infected.

## Discussion

We investigated 1021 COVID-19 inpatients diagnosed through RT-PCR in six different health facilities in Bangladesh. We believe that this study will shed light on the epidemiological aspects of COVID-19 during the pandemic. Although the study revealed facts that align with those established globally, some observations require further follow up and extensive studies in Bangladesh.

Our study has revealed that among the age groups, the 20–39 years cohorts showed the highest infection rate (54.7%) and in terms of gender, the prevalence of SARS-CoV-2 infection in males (74.9%) was three times more than that in females (25.1%). Several studies from across the globe have shown higher median age [[10], [11], [12]], mean age [13], or age group [10] for SARS-CoV-2 infection. We explained our observations in light of the demographic and socio-economic situations in Bangladesh, where 21–40 years is the most preponderant age group (https://www.cia.gov/library/publications/the-world-factbook/fields/341.html), and young and middle-aged people are mostly working and many need to leave home for work, and hence have higher odds of becoming infected [14]. Females, especially in rural Bangladesh, are mostly confined to household work, which might be a cause for less infection among this group.

Among the study participants, 38.33% had comorbidities. Diabetes was found to be the most prominent comorbidity (19.40%), and this fact was reflected in similar studies conducted in China and the UK [12,15]. Other noteworthy comorbidities were respiratory (8.40%) and cardiovascular (7.70%) diseases and the results obtained from our study aligned with trends in other countries in terms of high prevalence [10,11,15]. People who have a weak immune system are more susceptible to COVID-19. A number of studies reported that people with pre-existing conditions face a higher mortality rate when compared with people with no comorbidities. Comorbidities such as hypertension and diabetes reduce the immunity of a person significantly, thereby facilitating entry of the virus to lung cells using the angiotensin-converting enzyme-2 receptor [16,17].

As for the symptoms associated with COVID-19, fever (72.4%) and cough (55.9%) were the most prominent and were shared among cases (Table 2). Other clinical manifestations with significant association (p < 0.01) were breathing difficulty (25.90%) and chills (12.50%). Diarrhoea and gastrointestinal symptoms were also widely observed (19.80%) in this study; similar to results obtained during the MERS/SARS-CoV pandemics [18].

Of the 1021 COVID-19-positive cases in our study, 10.9% were asymptomatic, which is in accordance with the findings in South Korea and China [10,19]. Interestingly, our observation identified an upward trend for the increase in asymptomatic COVID-19 cases following April 2020 (Fig. 1). Although no systematic assessment has been performed on this aspect, our observation complies with the records and pronouncements of the health professionals and hospital registries in Bangladesh. For a better insight into the upward trend of asymptomatic COVID-19 cases, further studies are required including a larger data set. However, we hypothesize that as time progresses, more young people will be infected. It is known that asymptomatic COVID-19 is more common among younger people [20] and our data reflect this. Whether there were any molecular mechanisms involved in the process were beyond the scope of this study. We believe future research can be initiated based on this observation in our paper which might help to find the answer to this very interesting phenomenon. However, genetic diversity or new mutations might be responsible for asymptomatic cases [21,22].

The current study observed different biochemical parameters obtained from clinical specimens of the patients and categorized the results as low, normal and high compared with a reference value (Fig. 2A). A total of 219 (48%) symptomatic cases and 25 (39%) asymptomatic cases had a high level of D-dimer, establishing COVID-19-related coagulopathy, which is probably a manifestation of a profound inflammatory response [23]. For other parameters, a rise in ferritin, C-reactive protein, erythrocyte sedimentation rate and glucose levels were found in 37.92%, 36.08%, 27.37% and 36.48% of the COVID-19 cases, respectively. These findings agreed with results obtained from similar studies in China conducted on a sample size of 99 and 138 patients, respectively [24,25].

Our study revealed that 28.75% of patients, asymptomatic and symptomatic together, had a raised troponin level and it is assumed that this is owing to myocarditis, microangiopathy, myocardial infarction and cytokine storm [23,[26], [27], [28], [29]]. Elevated liver enzyme alanine transaminase was also found in 36.83% cases in this study, which is also in accordance with many other papers confirmed by review [30]. Although, a possible mechanism is yet to be confirmed, most of these cases reported long-term use of multiple drugs, which could have contributed to liver cell damage. Additionally, ischaemia and immune-mediated injury might be responsible for elevated liver enzymes in COVID-19-positive cases [30]. These observations suggest that patients diagnosed with COVID-19 should also undergo follow-up diagnosis procedures to check for chronic damage of the heart and liver.

Total cell count reports for COVID-19-positive individuals indicated that neutrophil count was elevated in 46.44% of patients. In contrast, reduced lymphocyte and platelet counts have been observed among 50% and 10% of COVID-19-positive individuals. This is in accordance with several other case series around the globe [23,[31], [32], [33], [34]]. As our study included individuals who had recovered from COVID-19, it is clear that these fluctuating parameters for the blood cell counts were not lethal; however, whether they were related to any severe symptoms prior to recovery requires further investigation. Our study did not investigate the level of cytokines or other inflammatory mediators in the patients. Further studies in this regard will be helpful in understanding the symptom severity and fatality among COVID-19 patients with respect to immune response mediators.

Human-to-human transmission of SARS-CoV-2 has been established as the major mode of spread and several reports provided evidence of transmission of COVID-19 by direct and indirect contact in hospital, family and community settings where interventions were not applied and basic health norms and distancing were not maintained [[35], [36], [37], [38]]. In our study, the majority of the cases (62%) reported indirect contact with confirmed cases, whereas 48.5% admitted to going out of their homes frequently before being infected and diagnosed (see Supplementary material, Table S1). It further shows that 50% of the respondents had close contact with confirmed cases and 40.6% had COVID-19-positive family members. It can be concluded from these data that community transmission is very common in Bangladesh and hence social distancing and other preventive measures should be fully implemented to prevent further deterioration.

Our study has also shed light on the relationship between the ABO blood groups and susceptibility to SARS-CoV-2 infection. A number of previous reports suggested that individuals with an A-positive (+ve) blood group have the highest risk of being infected while O(+ve) individuals have a comparatively lower risk rate [[39], [40], [41]]. Our study has shown that the majority of the cases belong to the B(+ve) blood group (35.4%), preceded by O(+ve) 28.2% and A(+ve) 20.3% (Table 1). However, this ratio is comparable to the population distribution of ABO blood groups (B+ve ∼34%, O+ve ∼30% and A+ve ∼26%) in Bangladesh [42], so we assume that the variation in the geographical prevalence of different blood groups might be the reason behind our findings.

An important part of our study was to assess and scrutinize the patients for post-COVID complications, both physical and mental (Table 3, and see Supplementary material, Table S2). Our study found that people with comorbidities have reported post-COVID complications such as mobility problems, pains and aches, anxiety and depression and indication of memory loss with a greater significance (see Supplementary material, Table S3). We also found that the vulnerability of recovered individuals towards post-COVID complications was linked to having comorbidities and being exposed to drugs for a prolonged period (Table S3). Although retrieved from a basic questionnaire, the results obtained from this study are statistically significant. Our findings are consistent with data obtained from similar studies in other countries [43,44]. In this study, no significant relationship between blood group and post-COVID-19 short-term outcome was found. This needs further study in a large number of patients.

This study involves an early epidemiological analysis of COVID-19 cases in Bangladesh, so it has some unavoidable limitations. First, the study only included inpatients from six selected hospitals providing treatment to COVID-19 patients in the respective areas, which narrows the sample size and diversity of the results. Second, because the data were collected over the telephone there are possibilities of information bias, interviewer bias and recall bias. Finally, an individual's health status and post-COVID-19 complications, depending on a 4-week recovery period, covers a short window of time. However, this study served the purpose of explaining the early post-COVID-19 health complications very well within the limited experimental scopes.

To date this is the most comprehensive study conducted in Bangladesh that assessed COVID-19 patients for both pre-recovery and post-recovery complications. Our data have reconfirmed some significant global observations with regard to the epidemiological and clinical aspects of the disease, but above all, the study has helped in addressing concerns that raised eyebrows including the role of ABO blood groups and bacillus Calmette–Guérin vaccination in COVID-19 susceptibility and progression. We anticipate that the outcomes of this study will work as a baseline for future studies in the same context.

## Conclusion

Despite some limitations, this is the most comprehensive study conducted in Bangladesh that assessed COVID-19 patients for both pre-recovery and post-recovery complications to date. Our data have reconfirmed some significant global observations with regards to the epidemiological and clinical aspects of the disease. Above all, the study has helped in addressing some concerns regarding COVID-19 susceptibility and progression in Bangladesh. This study found that individuals with comorbidities had more severe clinical implications acquired during their COVID-19 spells. The results obtained from this study could contribute to devising effective strategies for the provision of comprehensive health care to COVID-19 patients with comorbidities. We assume that the outcomes of this study will work as a baseline for future studies in the same context.

## Conflicts of interest

The authors have no conflicts of interest to report.
