# Supplementary material for: A multi-centre, cross-sectional study on coronavirus disease 2019 in Bangladesh: clinical epidemiology and short-term outcomes in recovered individuals
Source: New Microbes New Infect. 2021 Jan 8;40:100838. doi: 10.1016/j.nmni.2021.100838 (PMC7834423; doi:10.1016/j.nmni.2021.100838)
Supplement: Multimedia component 1 [file mmc1.doc]

**A multicentric, cross-sectional study on COVID-19 in Bangladesh: Clinical epidemiology and short-term outcomes in recovered individuals**

**Supplementary file-1**

| **Table S1** Contact history of SARS-COV2-infected patients | |
| --- | --- |
|  | N (%) |
| Having family member diagnosed with COVID-19 | 409 (40.6%) |
| Close contact with confirmed case | 493 (50%) |
| Indirect contact with confirmed case | 608 (62%) |
| Contact with individuals returning from abroad | 53 (5.4%) |
| Going outside frequently prior to getting infected | 466 (48.5%) |

| **Table S2** post-COVID complications of asymptomatic and symptomatic patients | | | | |
| --- | --- | --- | --- | --- |
|  | All patients,  (N=1,021) | Asymptomatic, (N=111) | Symptomatic, (N=910) | P value |
| Mobility problem | 179 (17.7) | 1 (0.9) | 178 (19.6) | 0.000 |
| Routine-activity weakness problem | 104 (10.7) | 3(2.7) | 101 (11.1) | 0.006 |
| Pain and aches | 319 (31.8) | 16 (14.4) | 303 (33.3) | 0.000 |
| Anxiety and depression | 230 (23.1) | 7 (6.3) | 223 (24.5) | 0.000 |
| Sleep disturbances | 312 (32) | 20 (18) | 292 (32.1) | 0.003 |
| Panic attack | 121 (12.4) | 6 (5.4) | 115 (12.6) | 0.024 |
| Concentration inability | 243 (24.4) | 10 (9) | 233 (25.6) | 0.000 |
| Memory loss | 195 (19.5) | 8 (7.2) | 187 (20.5) | 0.001 |

| **Table** S3 Adjusted odds ratios for predictors of post-COVID complications | | | | | | | | |
| --- | --- | --- | --- | --- | --- | --- | --- | --- |
|  | 1 | 2 | 3 | 4 | 5 | | 6 | 7 |
|  | Memory loss | Weakened attention span | Anxiety & depression | Mobility problem | Weakness | Sleep disturbance | | Pain & aches |
| Diabetes | — | — | — | — | — | — | | — |
| Cancer | — | — | 0.10*  (0.138) | — | — | — | | — |
| Cardiovascular disease | — | — | — | — | — | — | | — |
| Respiratory disease | 1.70**  (0.477) | 1.62*  (0.433**)** | — | — | — | 2.41***  (0.652) | | 1.70**  (0.445) |
| Kidney disease | — | — | — | — | — | — | | — |
| Liver disease | — | — | 9.76***  (6.643) | 6.06***  (3.499) | 5.07***  (2.957) | 2.88*  (1.701) | | — |
| Other chronic disease | — | — | 1.95**  (0.546) | — | — | — | | — |
| Prolonged medication use | 1.85***  (0.436) | 1.84***  (0.403) | 1.65**  (0.375) | — | — | 2.16***  (0.470) | | — |
| No. of observations | 902 | 898 | 894 | 905 | 863 | 872 | | 901 |
| All models are controlled for age, gender, BMI, vaccination. *** P<0.01, ** P<0.05, * P<0.10,  Parentheses include standard errors. Coefficients are reported if they are statistically significant | | | | | | | | |

| **Table S4** Comparison of Post-COVID complications among patients with most prevalent blood groups | | | | |
| --- | --- | --- | --- | --- |
|  | A+ (N=207) | AB+ (N=92) | B+ (N=360) | O+ (N=287) |
|  | N (%) | N (%) | N (%) | N (%) |
| Mobility problem | 37 (18.14) | 22 (23.91) | 53 (14.80) | 58 (20.42) |
| Routine-activity weakness problem | 23 (11.73) | 13 (14.77) | 35 (10.17) | 30 (11.07) |
| Pain and discomfort | 70 (34.31) | 30 (32.61) | 102 (28.65) | 99 (35.36) |
| Anxiety and depression | 48 (23.76) | 22 (23.91) | 89 (25.21) | 62 (22.30) |
| Sleep disturbances | 76 (37.81) | 37 (42.05) | 107 (31.10) | 74 (27.31) |
| Panic attack | 22 (11.06) | 11 (12.09) | 48 (13.91) | 33 (12.04) |
| Weakened attention span | 44 (21.78) | 24 (26.67) | 98 (27.61) | 65 (23.13) |
| Memory loss | 39 (19.12) | 18 (19.78) | 75 (21.19) | 52 (18.51) |
